# Basal Signalling Through Death Receptor 5 and Caspase 3 Activates p38 Kinase to Regulate Serum Response Factor (SRF)-Mediated MyoD Transcription

**DOI:** 10.5334/1750-2187-14-1

**Published:** 2020-05-08

**Authors:** Jason A. Ross, Brianna Barrett, Victoria Bensimon, Girish Shukla, Crystal M. Weyman

**Affiliations:** 1 Center for Gene Regulation in Health and Disease, Department of Biological, Geological, and Environmental Sciences, Cleveland State University, Cleveland, OH, US

**Keywords:** DR5, caspase 3, p38 kinase, SRF, MyoD

## Abstract

We have previously reported that stable expression of a dominant negative Death Receptor 5 (dnDR5) in skeletal myoblasts results in decreased basal caspase activity and decreased mRNA and protein expression of the muscle regulatory transcription factor MyoD in growth medium (GM), resulting in inhibited differentation when myoblasts are then cultured in differentiation media (DM). Further, this decreased level of MyoD mRNA was not a consequence of altered message stability, but rather correlated with decreased acetylation of histones in the distal regulatory region (DRR) of the MyoD extended promoter known to control MyoD transcription. As serum response factor (SRF) is the transcription factor known to be responsible for basal MyoD expression in GM, we compared the level of SRF binding to the non-canonical serum response element (SRE) within the DRR in parental and dnDR5 expressing myoblasts. Herein, we report that stable expression of dnDR5 resulted in decreased levels of serum response factor (SRF) binding to the CArG box in the SRE of the DRR. Total SRF expression levels were not affected, but phosphorylation indicative of SRF activation was impaired. This decreased SRF phosphorylation correlated with decreased phosphorylation-induced activation of p38 kinase. Moreover, the aforementioned signaling events affected by expression of dnDR5 could be appropriately recapitulated using either a pharmacological inhibitor of caspase 3 or p38 kinase. Thus, our results have established a signaling pathway from DR5 through caspases to p38 kinase activation, to SRF activation and the basal expression of MyoD.

## Introduction

The coordinate regulation of differentiation and apoptosis is essential for proper development and tissue homeostasis. This synchronous control serves two distinct functions. Firstly, in a few cell types, synchronous control of the differentiation and apoptotic processes is necessary because functional differentiation requires certain morphological events associated with the apoptotic phenotype [1]. Secondly, in many cell types, the signaling pathways controlling differentiation and apoptosis are intertwined to assure that either harmful cells or those generated in excess are removed in an efficient manner that does not elicit an immune response [2,3]. The formation of skeletal muscle utilizes this latter scenario that necessarily results in the distinct biological endpoints of either differentiation or apoptosis [4,5,6,7]. While the removal of excess cells is critical during development, it is potentially detrimental to regeneration or cell therapy. If blocking apoptosis while allowing differentiation is to be considered as a potential approach to increasing the efficacy of regeneration or cell therapy, then a thorough understanding of how these processes are coordinately regulated is imperative [8,9].

To this end, we have previosuly reported that the classically pro-apoptotic death receptor 5 (DR5)/FADD/caspase 8 pathway, in cooperation with increased levels of the pro-apoptotic Bcl2 family member PUMA, plays a role in the efficient apoptosis associated with skeletal myoblast differentiation [10,11,12]. Specifically, when myoblasts expressing a dominant negative DR5 (dnDR5) are switched from growth media (GM) to differentiation media (DM), caspase activation, Bid cleavage, and the ensuing apoptosis are severely impaired relative to parental myoblasts. However, unlike the PUMA pathway, the DR5/FADD/caspase 8 pathway is also critical to skeletal myoblast differentiation. The effect of the DR5/FADD/caspase pathway on differentiation is exerted in GM and results in decreased levels of MyoD mRNA and protein [13]. Thus, we designed experiments to delineate the signalling pathway blocked by dnDR5, and therefore engaged by DR5, that is responsible for maintaining MyoD mRNA, and thus protein, levels. Herein, we present data to indicate that basal signalling through DR5 and caspase 3 activates p38 kinase to regulate serum response factor (SRF)-mediated MyoD transcription.

## Methods

### Cells and cell culture

The growth 23A2 myoblasts and 23A2 myoblasts expressing dnDR5 have been reported previously [10]. The Z-DEVD-fmk caspase inhibitor (20 µM final treatment concentration; Calbiochem) and SB 203580 (3 µM treatment concentration; Sigma) were each dissolved in DMSO. Appropriate volumes of DMSO or methanol alone were added to control cultures and did not exceed 0.15% v/v.

### Chromatin immunoprecipitation

ChIP was performed following the protocol provided in the EZ-ChIP^TM^ kit (Millipore/Upstate) and as described in [13]. Cells were plated on 150 mm plates. The next day, cells were fixed in 0.5% formaldehyde for 10 minutes at room temperature. Formaldehyde was inactivated by the addition of .125 M glycine to the cells for 5 minutes at room temperature. Cells were then washed with ice cold PBS containing 5 mM Na Butyrate and 0.5 mM PMSF and pelleted by centrifugation at 1500 rpm for 5 minutes and then resuspended in 5 ml cold Cell Lysis Buffer (CLB: 60 mM KCl, 15 mM NaCl, 5 mM MgCl, 10 mM Tris pH 7.4, 300 mM sucrose, 0.1 mM EGTA, 0.1% NP-40, 5 mM Na Butyrate, 0.5 mM PMSF). Cells were sonicated once for 10 sec to ensure lysis of the plasma membrane. Isolated nuclei were washed once in 30 ml of CLB and once in 1 ml of cold Nuclei Digestion Buffer (Cell Lysis Buffer without NP-40 and PMSF). For MNase digestion, intact nuclei were resuspended in 125 μl of Nuclei Lysis Buffer (prewarmed to 37°C), digested with MNase (50 units/ml) at 37°C for 5 minutes, and terminated by 5 mM EDTA. An aliquot from each sample was assessed for sufficient chromatin fragmentation (500–1000bp) by gel electrophoresis. Samples were sonicated twice to ensure lysis of the nuclei prior to immunoprecipitation. The remaining steps of the immunoprecipitation were performed using the EZ ChIP™ Chromatin Immunopreipitation Kit (Upstate) per manufacturer’s instructions. Subsequently, anti-SRF (Santa Cruz) or appropriate IgG control (Sigma Aldrich) were added for immunoprecipitation. For each immunoprecipitation, 5 µg of the appropriate antibody was incubated with a precleared chromatin aliquot overnight at 4°C with rotation. The next day, protein A/G sepharose beads were added and incubated for 1 hour at 4° with rotation. The immunoprecipitates were pelleted, washed and the antibody-protein-DNA complex was eluted from bead by incubation in 100 mM NaHCO_3_ and 1%SDS. Following immunoprecipitation and elution, the eluent was treated with RNase A followed by reverse crosslinking by incubation at 65°C overnight. Protein was removed by addition of proteinase K and incubation at 45° for 2 hours. DNA was purified using mini columns provided by kit manufacturer. Purified DNA was amplified by specific primers [13] and PCR was performed under the following conditions: 1 cycle at 95° for 15 min, 40 cycles of 94° 1 min, 58° 1 min, 72° 1 min; and a final extension step at 72° for 5 minutes followed by analysis of melting curve. Data was normalized to the signal detected from the input of each sample. The fold enrichment of each target site was calculated as 2 to the power of the cycle threshold (cT) difference between input chromatin and ChIP samples.

### Western Analysis

Lysates were prepared and 100 μg were denatured and electrophoresed through denaturing polyacrylamide gels (10%) followed by electrophoretic transfer as previously described. For assessment of phospho-SRF and phospho-p38 relative to their cognate total protein levels, the same lysate was run on separate gels due to the utilization of a separate membrane blocking protocol for anti-phospho antibodies relative to the blocking protocol used for all other antibodies. For the following antibodies, each diluted 1:1000: from Santa Cruz; anti-SRF, from Cell Signalling; anti-p38, and from Abcam; anti-MyoD, membranes were blocked for one hour in 1 × TBS/0.1%NP40 with 10% newborn calf serum and 5% dry milk. For the following antibodies, each diluted 1:1000: from Cell Signalling, anti-phospho-p38 and anti-phospho-SRF, membranes were blocked for one hour in 1 × TBS/0.1%NP40 with 5% BSA. Western analysis using anti-actin (Sigma) or anti-hsp70 (BD Biosciences) served as loading and transfer controls (each diluted 1:30,000). All primary antibodies were incubated overnight at 4°C. Appropriate HRP-conjugated secondary antibodies, diluted 1:1000, were incubated with the membranes for one hour. After each incubation with antibody and prior to the addition of chemiluminescent substrate, membranes were washed five times in 1 × TBS (Tris- buffered saline pH 7.4) with 1% Tween 20. Membranes were then incubated with (SuperSignal West Pico Chemiluminescent Substrate: Thermo Scientific: #34078) for 60 seconds and bands were visualized using (Li-Cor Phospho-imager: Image Studio Ver. 2.1). Note that multiple Western analysis were run from the same set of samples to eliminate as much variablity as possible.

### Quantitative RT-PCR

Myoblasts were plated at equal density and the next day cultured as indicated in the figure legend. For quantitative RT-PCR, total RNA was prepared using 1 mL of Trizol (Invitrogen) reagent per 100 mm plate for lysis and following the manufacturer’s instructions. Five hundred ng of RNA was then used for a 20 µL SuperScript III RT (Invitrogen) reverse transcription reaction. Quantitative PCR (qPCR) was performed for MyoD as described [13] using a Bio-Rad DNA Engine Opticon 3 Real-Time PCR System using SYBR® Green Master PCR Mix according to the manufacturer’s instructions (Qiagen).

## Results

### Effects of dn-DR5 on signaling events regulating MyoD expression

**Figure 1:**
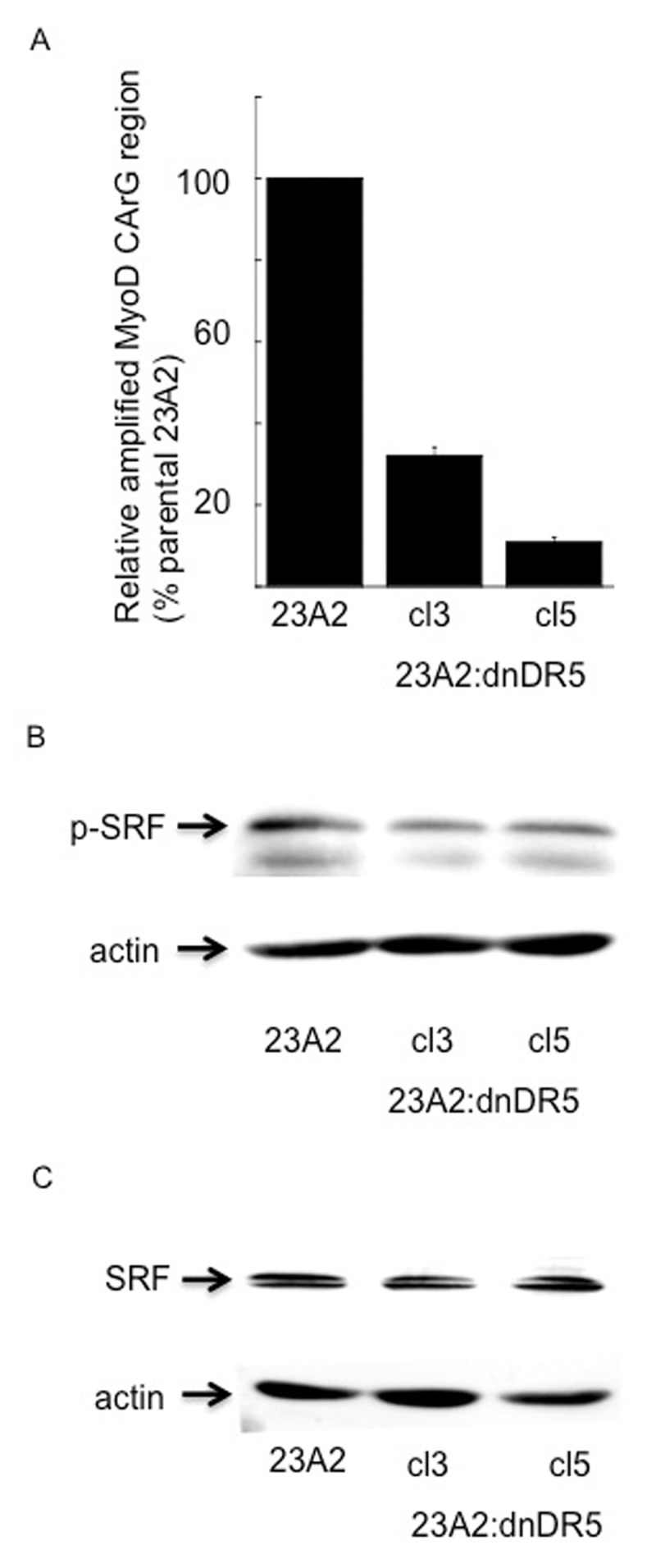
dnDR5 expression decreases SRF binding to the CArG box in the DRR of the MyoD enhancer as well as specific phosphorylation of SRF. In **(A)** chromatin from 1 × 107 cells was cross-linked and digested with MNase to a length between 500–1000 bp. Chromatin Immunoprecipitation was performed on each cell sample using EZ ChIPTM Chromatin Immunoprecipitation Kit (Upstate) per manufacturer’s instructions. Chromatin from 2 × 106 cells was immunoprecipitated as described in Methods. Quantitative PCR was used to assay for the relative levels of SRF binding near MyoD CArG element. Data was normalized to the signal detected from the input of each sample and presented as a percent of the signal obtained from parental 23A2 myoblasts. Error bars represent mean +/– SEM of triplicates. In **(B)** and **(C)**, equal cell numbers were plated and the next day lysates were prepared and subjected to SDS-PAGE. Western analysis was performed using anti-phospho SRF, anti-SRF or anti-β-actin (loading and transfer control) and visualized as described in Methods. Shown are the results of one experiment that are representative of three independent experiments for (B) and two independent experiments for (C).

SRF is the transcription factor known to drive the expression of MyoD in GM by binding the CArG box in the serum response element (SRE) of the distal regulatory region (DRR) of the MyoD gene [14]. To investigate the effect of dn-DR5 expression in skeletal myoblasts on the binding of SRF to this CArG box, we utilized chromatin immunoprecipitation (ChIP) analysis. The binding of SRF to the CArG box in the DRR of the MyoD gene in myoblasts expressing dn-DR5 was reduced by at least 70% when compared to levels of binding in parental control 23A2 myoblasts (Figure 1A). Specific phosphorylation of SRF is known to enhance the ability of SRF to specifically bind DNA [15]. These decreased levels of SRF bound to the CArG box as a consequence of dn-DR5 expression are not a consequence of an overall decrease in SRF levels, but rather correlate with decreased levels of this specifically phosphorylated SRF (Figure 1B and C).

**Figure 2:**
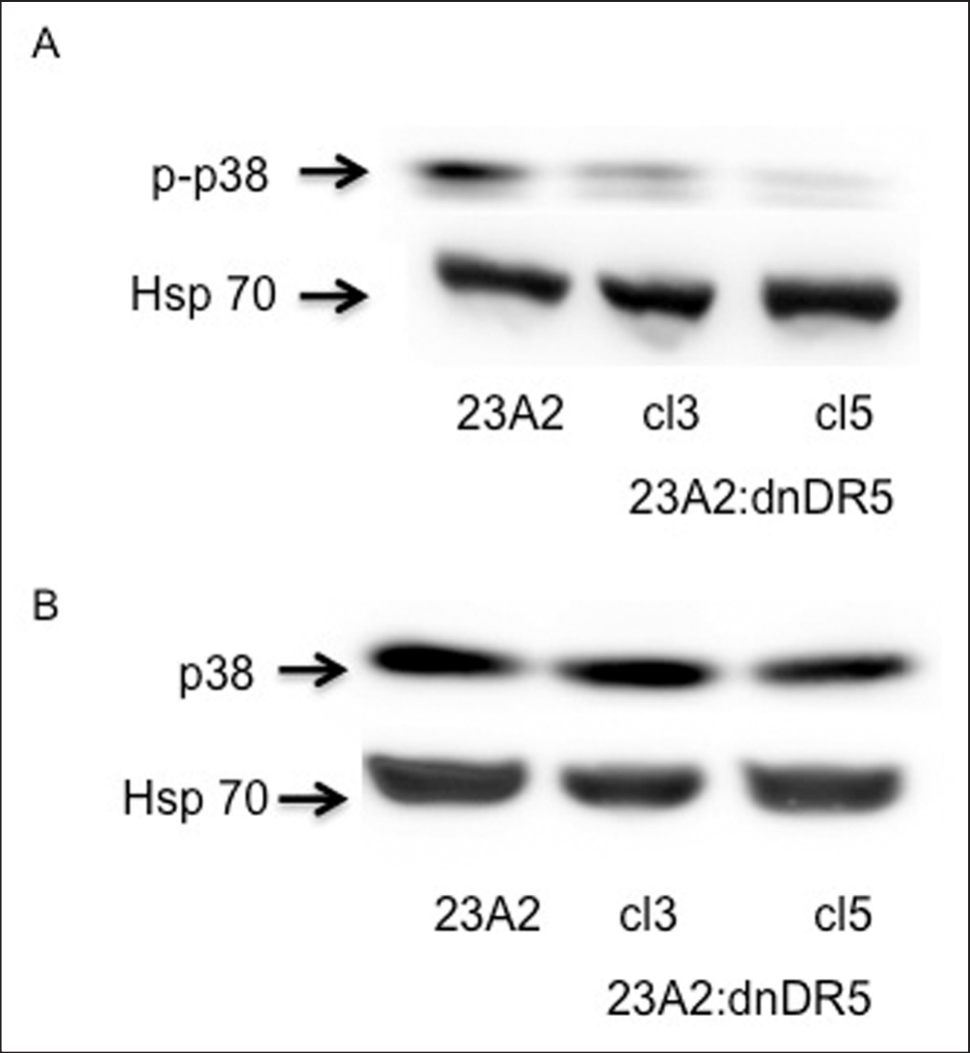
dnDR5 expressing myoblasts possess decreased levels of specifically phosphorylated p38. For each, equal cell numbers were plated and the next day lysates were prepared and subjected to SDS-PAGE. Western analysis was performed using anti-phospho p38 **(A)**, anti-p38 **(B)**, or anti-Hsp70 (loading and transfer control) and visualized as described in Methods. Shown are the results of one experiment that are representative of three independent experiments for (A) and two independent experiments for (B).

Several reports indicate that p38 kinase plays a pivitol role in regulating SRF phosphorylation and transcriptional activity [16,17,18,19]. We, therefore, utilized Western analysis to investigate the effect of dn-DR5 expression on the phosphorylation status of p38 indicative of activation [17]. We determined that lysates prepared from myoblasts expressing dn-DR5 possessed reduced levels of specifically phosphorylated p38, without a corresponding decrease in total p38, relative to lysates prepared from parental control myoblasts (Figure 2A and B).

### Effects of caspase 3 inhibition on phosphorylation of p38 and subsequent signaling events regulating MyoD expression

**Figure 3:**
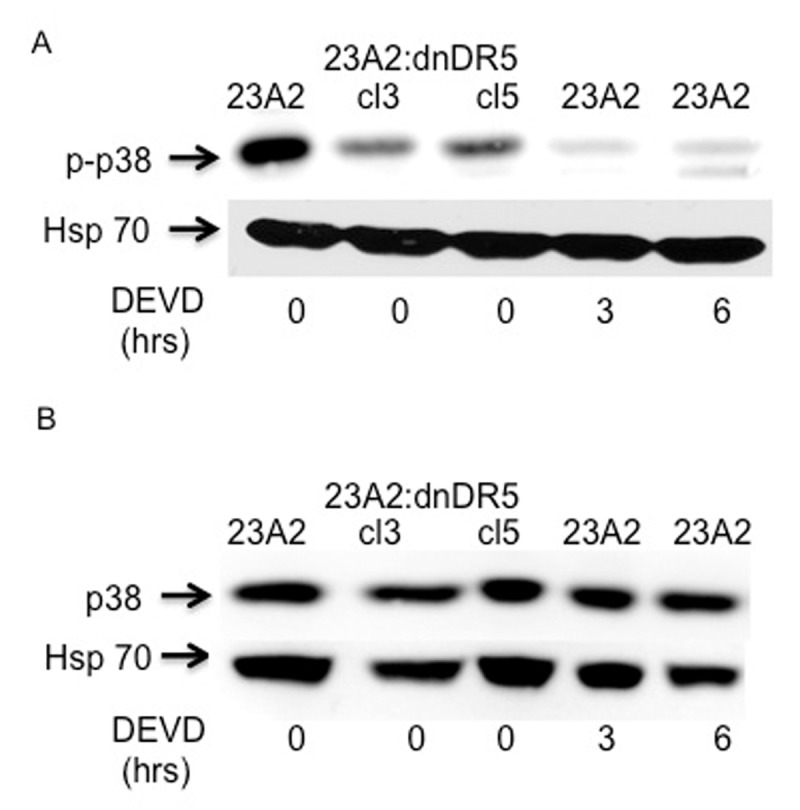
Myoblasts treated with a caspase 3 inhibitor possess decreased levels of specifically phosphorylated p38. For each, equal cell numbers were plated and the next day treated (or not) with DEVD-fmk as indicated in Methods. After the the indicated times, lysates were prepared and subjected to SDS-PAGE. Western analysis was performed anti-phospho p38 **(A)** or anti-p38 **(B)**. Western analysis using anti-Hsp70 served as the loading and transfer controls. Bands were visualized as described in Methods. Shown are the results of one experiment that are representative of three independent experiments for (A) and two independent experiments for (B).

**Figure 4:**
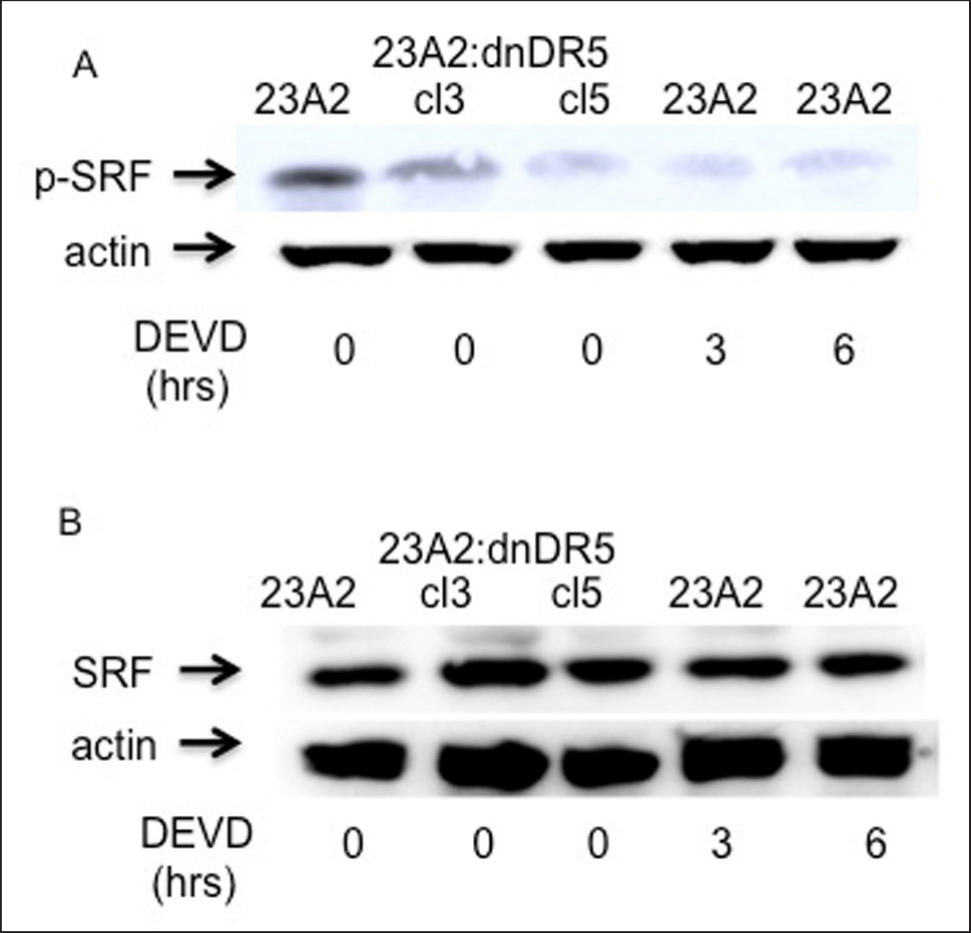
Myoblasts treated with a caspase 3 inhibitor possess decreased levels of specifically phosphorylated SRF. For each, equal cell numbers were plated and the next day treated (or not) with DEVD-fmk as indicated in Methods. After the the indicated times, lysates were prepared and subjected to SDS-PAGE. Western analysis was performed anti-phospho pSRF **(A)** or anti-SRF **(B)**. Western analysis using anti-Hsp70 served as the loading and transfer controls. Bands were visualized as described in Methods. Shown are the results of one experiment that are representative of three independent experiments for (A) and two independent experiments for (B).

**Figure 5:**
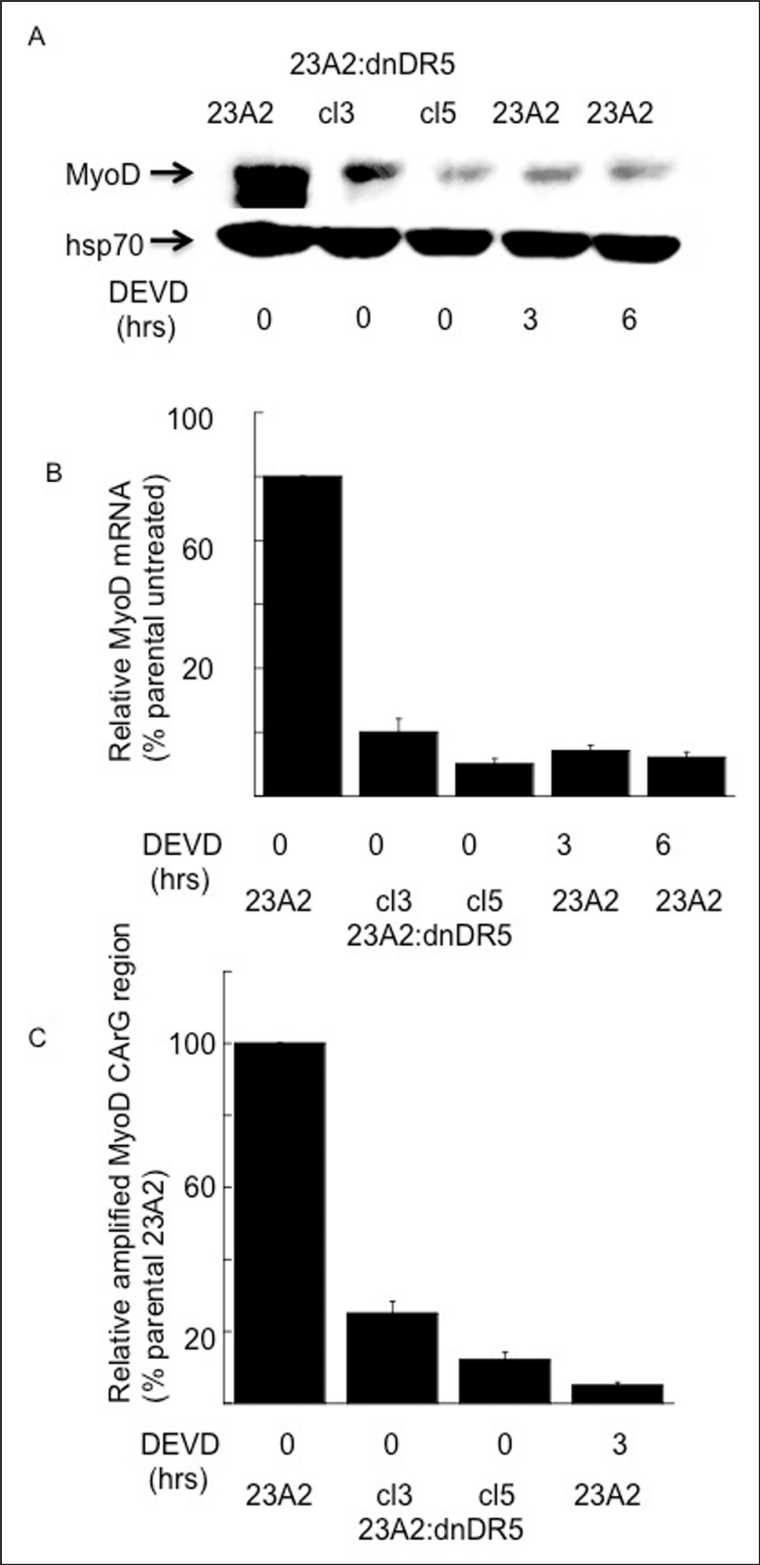
Myoblasts treated with a caspase 3 inhibitor possess decreased levels of MyoD protein, mRNA and SRF binding to the CArG box in the DRR of the MyoD enhancer. For each, equal cell numbers were plated and the next day treated (or not) with DEVD-fmk for the indicated times. In **(A)** Western analysis was performed using anti-MyoD or anti-Hsp70 (loading and transfer control) and visualized as described in Methods. Shown are the results of one experiment that are representative of three independent experiments. In **(B)** quantitative RT-PCR was used to assay for the relative levels of MyoD mRNA in total RNA samples derived from the indicated cell cultures. The Ct values for the MyoD PCR product were normalized to the Ct values for a β-actin product, run in parallel, as described in Methods. Error bars represent mean +/– SEM from triplicates. In **(C)**, ChIP analysis was perfomed as described in the Figure 1 legend.

Phosphorylation and activation of p38 kinase can occur in response to a multitude of signaling events, including caspase 3 mediated cleavage of several upstream kinases [17]. We have previosuly reported that expression of dn-DR5 in skeletal myoblasts results in decreased basal caspase 3 activation [13]. As a second approach to confirm that basal signaling from caspase 3 through p38 kinase results in phosphorylated SRF and subsequent maintenance of MyoD expression, we next treated parental myoblasts with the caspase 3 inhibitor DEVD-fmk. Treatment of parental myoblasts with DEVD-fmk for either 3 or 6 hours resulted in decreased levels of specifically phosphorylated p38 kinase (Figure 3A) without a corresponding decrease in total p38 (Figure 3B). Moreover, treatment of parental myoblasts with DEVD-fmk for either 3 or 6 hours resulted in decreased levels of specifically phosphorylated SRF (Figure 4A) without a corresponding decrease in total SRF (Figure 4B). Finally, treatment of parental myoblasts with DEVD-fmk resulted in decreased levels of MyoD protein (Figure 5A), a corresponding decrease in MyoD mRNA (Figure 5B) and SRF binding to the CArG box in the DRR of the MyoD enhancer (Figure 5C). In each aspect measured, caspase 3 inhibition mimicked the effect of dn-DR5 expression.

### Effects of p38 kinase inhibition on phosphorylation of SRF and subsequent signaling events regulating MyoD expression

**Figure 6:**
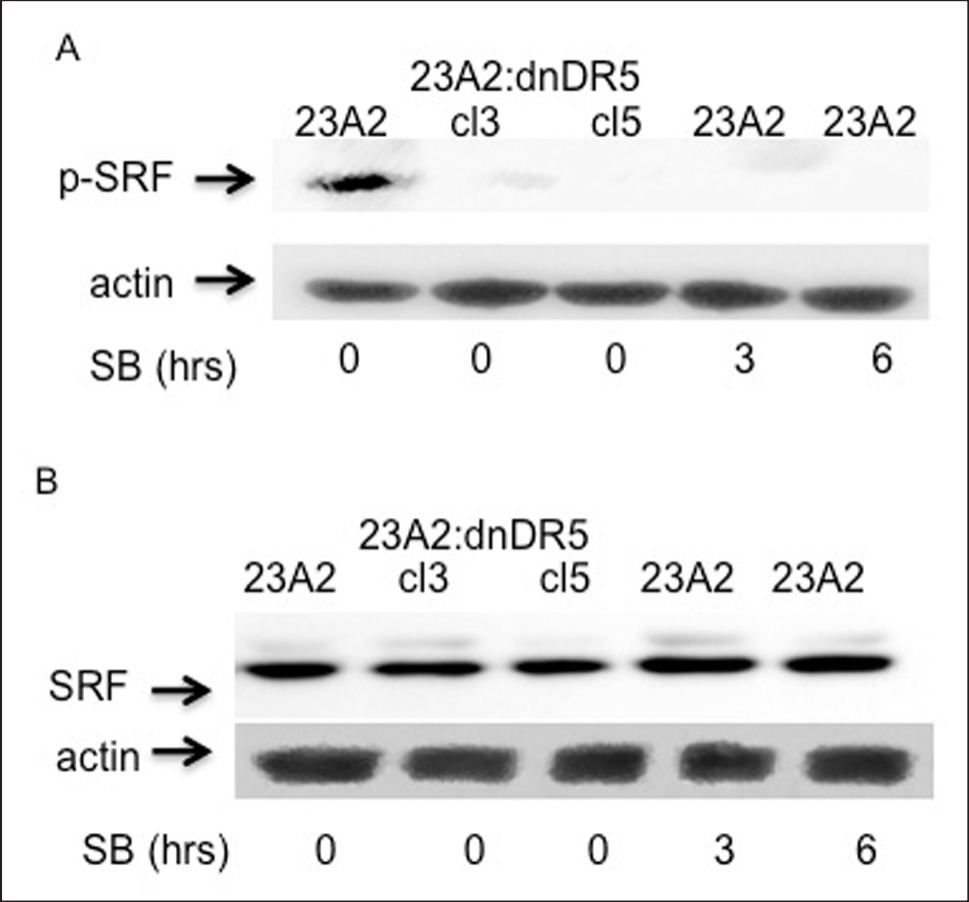
Myoblasts treated with a p38 inhibitor possess decreased levels of specifically phosphorylated SRF. For each, equal cell numbers were plated and the next day treated (or not) with SB 203580 for the indicated times. In **(A)** and **(B)**, Western analysis was performed using anti-phospho SRF or anti-SRF, respectively with anti-actin serving as the loading and transfer control. Bands were visualized as described in Methods. Shown are the results of one experiment that are representative of three independent experiments for (A) and two independent experiments for (B).

**Figure 7:**
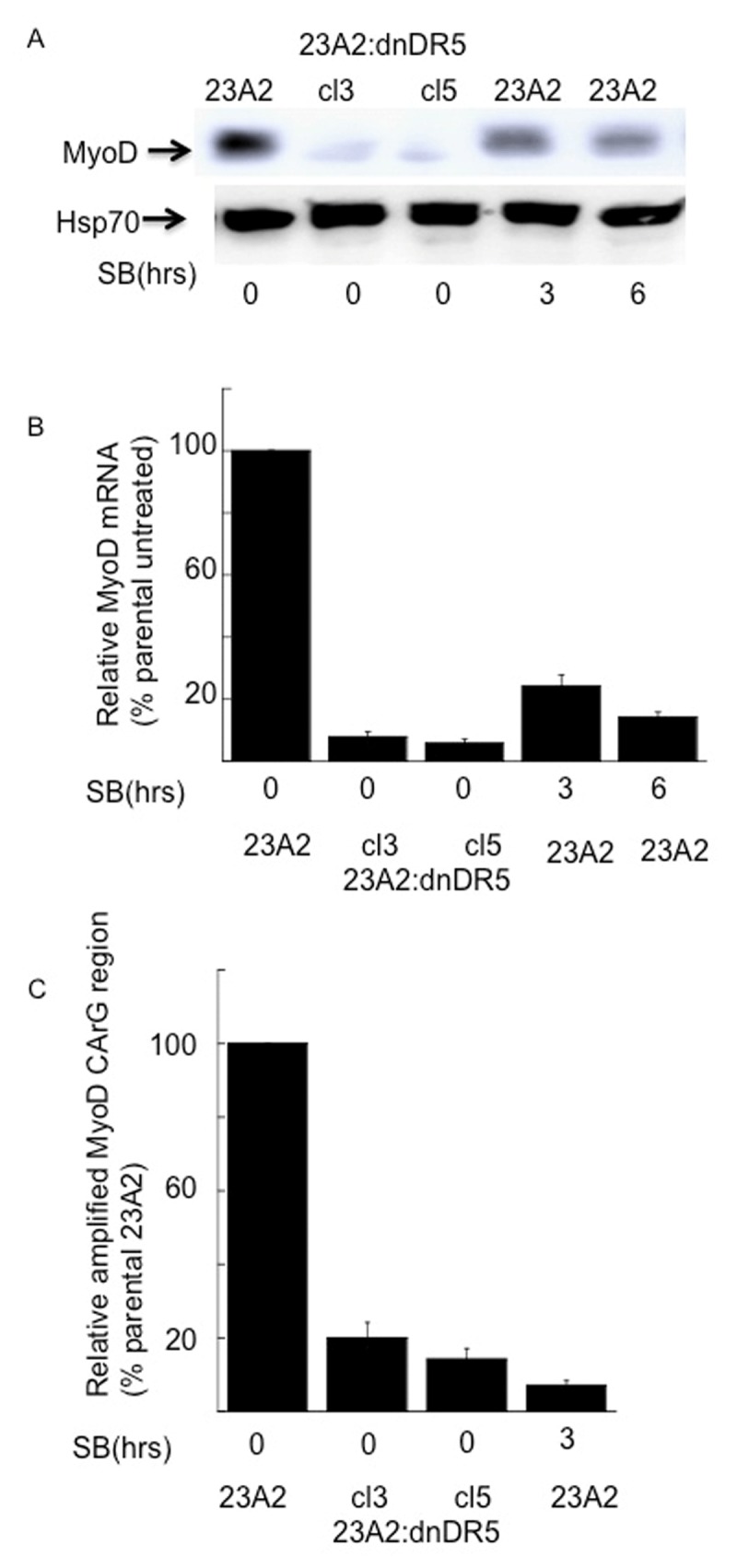
Myoblasts treated with a p38 inhibitor possess decreased levels of MyoD protein, mRNA and SRF binding to the CArG box in the DRR of the MyoD enhancer. For each, equal cell numbers were plated and the next day treated (or not) with SB 203580 for the indicated times. In **(A)**, Western analysis was performed using anti-MyoD or anti-Hsp70 (loading and transfer control) and visualized as described in Methods. Shown are the results of one experiment that are representative of three independent experiments. In **(B)**, quantitative RT-PCR was used to assay for the relative levels of MyoD mRNA as described in the legend to figure 5. In **(C)**, ChIP analysis was perfomed as described in the Figure 1 legend.

Finally, we treated parental myoblast with the p38 inhibitor SB 203580. Lysates prepared from parental myoblasts treated with SB 203580 for either 3 or 6 hours possessed decreased levels of phosphorylated SRF without a decrease in total SRF (Figure 6A and B). This decreased level of phosphorylated SRF induced by treatement with SB 203580 correlated with a corresponding decrease in MyoD protein and mRNA (Figure 7A and B, respectively) and decreased levels of SRF binding to the CArG box in the DRR of the MyoD enhancer (Figure 7C). In each aspect measured, p38 inhibition mimicked the effect of dn-DR5 expression.

**Figure 8:**
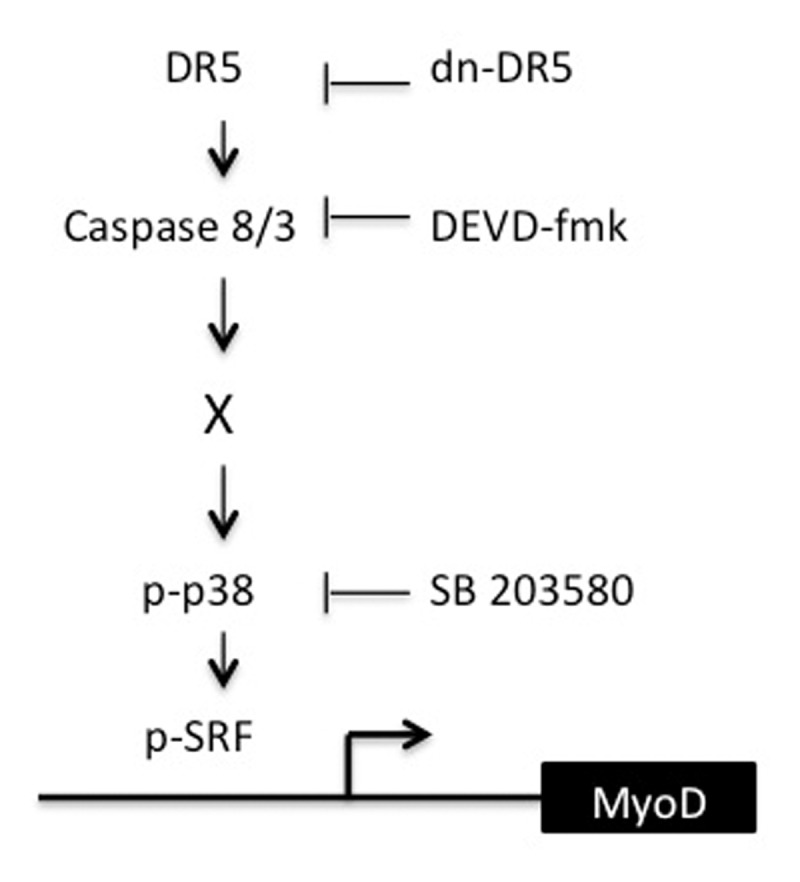
Model depicting the signalling pathway from DR5 to SRF-mediated transcription of MyoD. Basal signaling from DR5 to capases 8 and 3 results in specifically phosphorylated p38. Active p38 in turn leads to specifically phosphorylated SRF, SRF binding to the CArG box in the DRR of the MyoD enhancer, and expression of MyoD.

Direct confirmation that a decrease in the binding of phosphorylated SRF to the DRR awaits, at a minimum, the development of an anti-phospho SRF antibody that can be utilized for ChIP analysis. Nonetheless, taken together, these data still indicate that a decrease in caspase 3 activity, such as that detected upon expression of dn-DR5 in skeletal myoblasts [13], is sufficient to result in decreased phosphorylation and activation of p38 kinase, decreased levels of phosphorylated SRF, decreased binding of SRF to the CArG box in the DRR of the MyoD enhancer and decreased levels of MyoD expression (Figure 8/model).

## Discussion

A thorough molecular understanding of the coordinate regulation of differentiation and apoptosis is pre-requisite to the manipulation of these biologically distinct endpoints as a therapeutic approach. For instance, manipulation of the apoptotic pathway, without affecting the differentiation pathway, requires identification of molecules unique to the apoptotic pathway. To this end, we have previously reported that the DR5 pathway plays a critical role in both the apoptosis and the differentiation of skeletal myoblasts. In skeletal myoblasts, the DR5 proximal molecules responsible for executing both the differentiation and the apoptotic pathways is caspase 8 signaling through caspase 3. The distinction between signaling that allows for differentiation and signaling that induces apoptosis resides in the level of activation. Specifically, elevated signaling by DR5 in differentiation medium (DM) contributes to the apoptosis that occurs in a subset of myoblasts [10], while basal signaling from DR5/FADD/caspases in growth medium (GM) is necessary to maintain the level of MyoD expression and thus allow for the differentiation of the majority of myoblasts when cultured in DM [13]. Recently, the importance of widespread basal caspase signaling has been reported in whole animal studies [20,21].

A plethora of examples of non-apoptotic roles for apoptotic molecules have been reported. However, the vast majorities of these examples are for Bcl2 family members or caspases and rarely have the non-apoptotic signaling events downstream of the apoptotic molecules been elucidated [22,23,24]. The DR5 pathway has been implicated in the non-apoptotic role of inducing intestinal cell differentiation, but again the downstream signaling molecules were not determined [25]. With respect to myogenesis, a role for caspase-3 has been documented at several steps. Firstly, caspase-3 activity is required to remove Pax7, allowing myoblast stem cells to switch from self-renewal to differentiation competence [26]. Secondly, after myoblasts have been switched from GM to DM, caspase-3 mediated CAD (caspase-activated DNase) activation plays a role in the chromatin modification and subsequent expression of p21, one of the key molecules required for cell cycle exit [27]. Further, in DM, caspase-3 activation has been linked to the subsequent activation of p38 [28]. Multiple roles for p38 signaling during skeletal myoblast differentiation has also been well characterized [29]. These elegant studies carefully documented the molecular effects of caspase-3 and p38 signaling that contribute to the differentiation process in skeletal myoblasts. However, these studies did not address the mechanism(s) responsible for caspase-3 activation. Moreover, these studies were focused on signaling events that occur once myoblasts were cultured in DM.

Our studies were focused on signaling that occurs in GM. Herein, we idenitfy the molecules responsive to basal DR5 signaling in GM that serve to maintain MyoD expression and allow for subsequent differentiation in DM. Specifically, we document that basal DR5/caspase signaling in GM leads to constitutive phosphorylation and activation of the p38 kinase which in turn phosphorylates and activates SRF, driving the expression of MyoD. The significance of SRF activity in driving MyoD expression in skeletal myoblasts in both GM and DM has been well documented [14,30]. In DM, SRF activity has been shown to be regulated by RhoA [31]. Given that SRF is by definition serum responsive via Ras pathway signaling to the ternary complex factor (TCF) [32], further exploration of other mechansims contributing to the activation of SRF in GM has not been exhaustively addressed. Our data support a model whereby basal DR5 signaling through caspase-mediated activation of p38 plays a role in maintaining SRF activation and subsequent MyoD expression in GM.
